# Kaposi sarcoma herpesvirus pathogenesis

**DOI:** 10.1098/rstb.2016.0275

**Published:** 2017-09-11

**Authors:** Giuseppe Mariggiò, Sandra Koch, Thomas F. Schulz

**Affiliations:** 1 Institute of Virology, Hannover Medical School, Carl Neuberg Strasse 1, 30625 Hannover, Germany; 2 German Centre for Infection Research, Hannover-Braunschweig site, Hannover, Germany

**Keywords:** KSHV, viral infection, KSHV-related diseases, aberrant angiogenesis, DNA damage response, innate immune evasion

## Abstract

Kaposi sarcoma herpesvirus (KSHV), taxonomical name human gammaherpesvirus 8, is a phylogenetically old human virus that co-evolved with human populations, but is now only common (seroprevalence greater than 10%) in sub-Saharan Africa, around the Mediterranean Sea, parts of South America and in a few ethnic communities. KSHV causes three human malignancies, Kaposi sarcoma, primary effusion lymphoma, and many cases of the plasmablastic form of multicentric Castleman's disease (MCD) as well as occasional cases of plasmablastic lymphoma arising from MCD; it has also been linked to rare cases of bone marrow failure and hepatitis. As it has colonized humans physiologically for many thousand years, cofactors are needed to allow it to unfold its pathogenic potential. In most cases, these include immune defects of genetic, iatrogenic or infectious origin, and inflammation appears to play an important role in disease development. Our much improved understanding of its life cycle and its role in pathogenesis should now allow us to develop new therapeutic strategies directed against key viral proteins or intracellular pathways that are crucial for virus replication or persistence. Likewise, its limited (for a herpesvirus) distribution and transmission should offer an opportunity for the development and use of a vaccine to prevent transmission.

This article is part of the themed issue ‘Human oncogenic viruses’.

## 1. History

First described by Moritz Kaposi in 1872 as ‘idiopathic multiple pigmented sarcoma of the skin’ occurring mainly in elderly males of Mediterranean origin [1], Kaposi sarcoma (KS) has long been considered an unusual neoplasm. Its clinical manifestations can range from slowly progressive and confined to the skin of the legs to aggressive, involving several visceral organs. In Africa, particularly East and Central Africa, it was first noted long before the arrival of HIV and reported to also occur in children [2,3]. Although a very rare tumour in Western countries before 1980, its incidence suddenly increased dramatically with the arrival of HIV/AIDS when it became one of the first AIDS-defining malignancies.

Careful epidemiological studies soon showed that not all people with HIV/AIDS were equally affected by this tumour. While most common among HIV-infected men who have sex with men, it hardly ever occurred in HIV-infected men suffering from haemophilia and who had contracted HIV through contaminated factor VIII preparation in the 1980s before regular testing of blood and plasma donations for HIV had begun. KS was also rare in those who had contracted HIV through blood donations and among HIV-infected women [4]. These observations, together with a further detailed epidemiological analysis of the behavioural risk factors among people with HIV/AIDS, led to the conclusion that KS was most likely caused by a new sexually transmissible infectious agent that appeared to be transmitted independently of HIV. These predictions led to attempts in several laboratories to identify the putative transmissible cause of KS and eventually to the discovery, by Yuan Chang, Patrick Moore and colleagues, of sequence fragments belonging to a new human herpesvirus in biopsies of AIDS-associated KS [5]. Termed Kaposi sarcoma-associated herpesvirus (KSHV) by its discoverers, and classified taxonomically as human gamma herpesvirus 8 and a member of the γ_2_-herpesviral lineage in the subfamily of γ-herpesviruses, this new herpesvirus was soon shown to also be the cause of two B-cell malignancies, primary effusion lymphoma (PEL) [6] and the plasma cell variant of multicentric Castleman's disease (MCD) [7]. In addition, some cases of plasmablastic lymphoma [8,9], and polyclonal plasmacytic lymphoproliferations, which can arise from MCD tumours [10–12], were also be found to be positive for KSHV. Furthermore, several cases of bone marrow failure, with or without accompanying haemophagocytic syndrome [13–18] as well KSHV-associated cases of hepatitis [14,19] have been described.

During the following years, a substantial body of epidemiological and experimental evidence quickly accumulated to prove the causative role of KSHV in KS, PEL and MCD. As a result, KSHV was classified as a class I carcinogen by IARC/WHO in 2009 [20,21].

## 2. Epidemiological aspects and origin of KSHV

A thorough review of the wealth of published epidemiological studies on the geographical distribution and transmission of KSHV is beyond the scope of this article and we therefore only highlight the most important aspects below. For a more extensive review of the epidemiology of KSHV, the reader is referred to reference [21].

### (a) Geographical distribution

Compared with other human herpesviruses, which, with the exception of HSV-2, characteristically infect the majority of healthy individuals in most geographical regions, KSHV shows a very unusual geographical distribution. It is highly prevalent (greater than 50% seroprevalence rates) only in sub-Saharan Africa, moderately common around the Mediterranean basin (seroprevalence rates of 3–20%, depending on the geographical location sampled and the serological assays employed) and in some countries in South America, but infrequent (less than 10% seroprevalence) in most other parts of the world (for a summary of published studies, see [21]). There is, however, evidence of increased KSHV prevalence rates, or the presence of particular KSHV genotypes, in certain ethnic groups, such as the Uighur population in the Xinjiang region of China [22], people of Jewish descent [23], some native American populations [24,25] and the Buryat population in southern Siberia [26].

### (b) Transmission

In the general population, KSHV appears to be transmitted mainly during childhood, with saliva being considered the most important vehicle of transmission [27–31]. Several studies show that KSHV can be transmitted within families, from mother to child as well as among siblings [30,32–35].

As originally predicted by epidemiological studies looking for risk factors for KS in patients with HIV/AIDS (see above), KSHV can also be sexually transmitted [36–39] (for further references, see [21]). This seems to play a particularly important role in individuals at increased risk for contracting other sexually transmitted diseases. It is thought that saliva also represents the most important vehicle during sexual transmission, but KSHV has also been detected, inconsistently and in low copy numbers, in semen [40,41].

There is also some evidence for KSHV transmission via blood transfusion [42–44] and through injecting drug use [45,46], although sexual transmission as a confounding factor has been difficult to eliminate for the latter. Transmission in the context of the transplantation of solid organs can occur [14,19,47–50], and in some instances cells in a KS tumour of a transplant recipients have been shown to be of organ donor origin, suggesting that KSHV-infected endothelial cells had been transmitted in the transplanted organ and grown into a KS tumour in the transplant recipient [51]. However, many cases of post-transplant KS occur in recipients who were already KSHV seropositive at the time of transplantation [47,49], suggesting that reactivation of a pre-existing KSHV infection is frequently responsible for the development of post-transplant KS.

### (c) Origin and evolution of KSHV

KSHV (taxonomical name: human gammaherpesvirus 8) is classified as one of nine species in the genus *Rhadinovirus* of the subfamily *Gammaherpesvirinae* in the family *Herpesviridae* (www.ictvonline.org). The other ICTV-classified herpesvirus species in the genus *Rhadinovirus* are found in Old and New World monkeys, rodents and cattle. In addition, sequence fragments or nearly complete sequences of related rhadinoviruses have been documented in most Old World primate species, including the great apes gorilla and chimpanzee; these viruses appear to fall into two lineages, provisionally termed RV1 and RV2 [52–58]. These observations suggest that KSHV, like all other herpesviruses, has co-evolved with its host species over a very long time span. This notion is further supported by the fact that minor sequence variation (less than 3%) throughout most, and more extensive sequence variation in the two genes at either end, of the of the KSHV genome seem to have evolved in different geographical regions, suggesting coevolution with human populations [24,25,56,59–64]. As in the case of other herpesviruses, there is also evidence of recombination as a driving force in the evolution of KSHV genomes. This is most dramatically illustrated by the existence of three highly distant variants of the K15 gene at the ‘right’ end of the viral genome, which are thought to have resulted from recombination events with currently unknown KSHV precursors that could possibly have belonged to rare human or no longer existing hominid populations [25,60,63].

Given this coevolution of KSHV with human populations, the fact that it is today infrequent in many geographical areas still awaits a satisfactory explanation. Suggestions include host genetic polymorphisms that contribute to the maintenance of KSHV in endemic populations. In support of this possibility, the existence of a recessive gene on chromosome 3p22 predisposing to KSHV infection in childhood in an endemic population has been predicted [65,66]. Environmental factors such as plant chemicals that promote the reactivation of KSHV in infected individuals and thereby the spread in the community have also been invoked [67]. In addition, there is now increasing evidence that KSHV transmission in childhood in Africa is associated with parasitic infections, including malaria [68–70].

## 3. Pathogenesis

### (a) KSHV-associated tumours depend on the presence/expression of KSHV

Clinical, histopathological and experimental observations indicate that the continued presence of KSHV in tumour cells is required to maintain tumour growth. The practical implication of this conclusion would be that targeting the virus, or some of its proteins, could represent a successful approach to therapy (see below).

**Figure 1.**
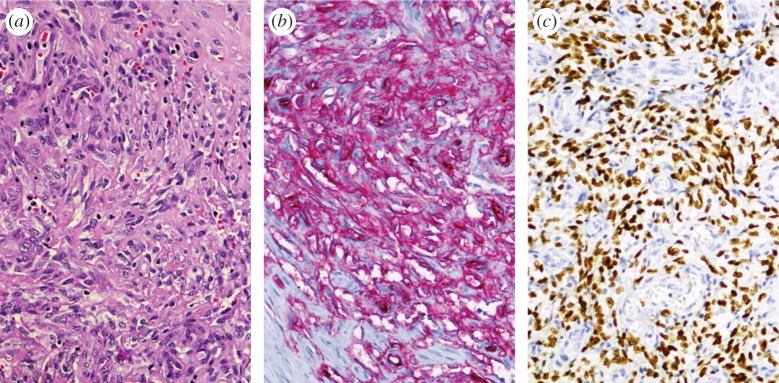
Histology of a KS tumour infiltrating a lymph node (250× magnification). (*a*) HE staining showing the typical histological features such as elongated spindle cells, abnormal vessels with thinned epithelium and extravasated erythrocytes. (*b*) Immunohistochemistry staining for CD34 to indicate the endothelial origin of the spindle cells. (*c*) Immunohistochemistry staining for LANA, showing tumour cells with a latent KSHV infection.

In all KSHV-associated tumour entities (KS, PEL, MCD and plasmablastic lymphoma) tumour cells express the latency-associated nuclear antigen (LANA), and this is used for diagnostic purposes to show the presence of KSHV-infected cells in these tumours by immunohistochemistry (figure 1). In the case of KS, the number of LANA-expressing cells can be variable, with some tumours only showing a small proportion of LANA-expressing cells while in others most tumour cells express LANA. Immunohistochemistry staining for latent and lytic KSHV proteins as well as transcriptional profiling of KS tumours indicates that some tumours show a restricted pattern of viral gene expression, which is largely limited to the genes for LANA, the viral homologues of a D-type cyclin (vCYC), the viral FLICE inhibitory protein (vFLIP), and the viral micro-RNAs (miRNAs), in addition to K1, K15 and the viral G-protein-coupled receptor homologue (table 1), while in others additional viral genes belonging to the lytic (productive) replication cycle may be expressed [216–218]. KS tumours are in most cases not monoclonal and different KS nodules in the same individual may have independent clonal origins [219–221]. In addition, KS in transplant recipients can regress following the reduction of immunosuppressive therapy and KS lesions in AIDS patients often respond to antiretroviral combination therapy. However, in a proportion of AIDS patients KS may persist, or reappear, in those with well-controlled HIV viral loads (see below).

**Table 1.** Contribution of selected KSHV proteins or miRNAs to viral life cycle and pathogenesis.

KSHV protein or RNA	function in viral life cycle	role in KSHV pathogenesis	references
K1	regulation of KSHV lytic replication; activation of PI3 K/Akt and MEK/Erk pathways; modulation of B-cell antigen receptor	increases angiogenesis and invasiveness of KHSV-infected endothelial cells and may contribute to increased vascular permeability; overexpression in transgenic mice shows oncogenic/transforming properties	[71–83]
K3	E3 ubiquitin ligase, downregulates MHC I		[84,85]
K5	E3 ubiquitin ligase, downregulates MHC I, ICAM-1, B7.2, BST/tetherin		[85–88]
K15	regulation of KSHV lytic replication; modulation of B-cell receptor-dependent signalling; activation of PLCγ1, MEK/Erk, JNK and NF-κB-dependent signalling	activation of angiogenesis and invasiveness in KSHV-infected endothelial cells; induction of inflammatory cytokines	[61,82,89–95]
kaposin A		promotion of proliferation of KSHV-infected endothelial cells; regulation of integrin-dependent cell adhesion and induction of glutamate receptor 1 expression	[96–98]
kaposin B	modulation of p38 signalling cascade by interaction with MK2 to stabilize cytokines and Prox-1 mRNAs; promotion of STAT3 phosphorylation	promotion of RhoA-dependent stress fibre formation, motility and angiogenesis of endothelial cells	[99–102]
LANA	major viral protein for the persistence of KSHV infection, replication of latent episomes and their distribution to daughter cells during mitosis; promotion of KSHV lytic reactivation by non-canonical cytoplasmic isoforms	inhibition of p53, p73 and pRB activity; redistribution of GSK3β and regulation of c-Myc and β-catenin; extension of the lifespan of KSHV-infected cells	[103–116]
miRNAs	miR-K12–1 and miR-K12–3 modulate NF-κB signalling to repress lytic reactivation; miR-K12-1 targets p21; miR-K12-5 reduces Rta expression to maintain latency; miR-K12-7 reduces expression of natural killer (NK) cell ligand MICB; miR-K12-11 (orthologue of miR-155) attenuates TGF-β signalling and modulates B-cell maturation	contribution of miR-K12-11 to B-cell expansion and transformation of rat mesenchymal precursor cells; contribution of miR-K12-6 and miR-K12-11 to KSHV-induced endothelial cell differentiation by reduction of the lymphatic endothelial cell-specific transcription factor MAF	[117–129]
vCYC	regulation of cell cycle; involved in latency control	promotion of RB-dependent S phase entry; induction of DDR signalling and oncogene-induced senescence (OIS), abrogation of contact inhibition of latently infected cells	[130–136]
vFLIP	activation of NF-κB cascade; contribution to latency and persistence by inhibition of lytic reactivation; anti-apoptotic and anti-autophagy function; induction of interferon (IFN)-inducible cellular genes	contribution to KS spindle cell formation and PEL cell survival; antagonistic role on vCYC-induced OIS, induction of Notch signalling-dependent IL6 expression; involved in KSHV-induced differentiation of endothelial cells and in EndMT; involved in B-cell differentiation; induction of PRC2-complex-mediated epigenetic changes; contribution to inflammatory infiltrate in KS lesions	[110,137–154]
vGPCR	regulation of KSHV lytic reactivation; downmodulation of TLR4 expression	cancerogenic and angioproliferative properties in transgenic mice; contribution to KS development by promotion of VEGF and angiopoetin expression; activation of PI3 Kγ/Akt/mTOR pathway; modulation of Notch-mediated cascade; promotion of KSHV-induced endothelial cell differentiation and EndMT	[139,151,155–166]
vIL6	activation of gp130 in the absence of IL6Rα; promotion of B-cell proliferation; induction of VEGF- and IL6-mediated angiogenesis; intracellular Notch signalling-dependent expression	promotion of PEL cell proliferation; contribution to VEGF- and IL6-mediated vascular permeability and to the pathogenesis of MCD and PEL; correlation of vIL6 levels with disease activity in MCD and inflammation; contribution to lymphatic endothelial cell differentiation of KSHV-infected blood vascular endothelial cells	[167–182]
vIRF1	modulation of cGAS/STING pathway; inhibition of vIRF3-mediated CBP/p300 recruitment; repression of IFN-inducible genes and MHC expression	modulation of apoptotic signalling by inhibition of p53 function and interacting with Bim, Bid and GRIM19; suppression of TGF-β/Smad signalling pathway; inhibition activation-induced cell death (AICD) by modulation of CD95 pathway	[183–191]
vIRF2	prevention of PKR activation and cellular IRF/STAT1-dependent IFN type I/III responses	inhibition of AICD by prevention of CD95 L expression	[192–194]
vIRF3	modulation of cellular IRF-mediated IFN type I response; inhibition of NF-κB activation by interaction with IKKβ; disruption of PML nuclear bodies and decrease of MHC II expression	modulation of p53 function and inhibition of PKR-mediated apoptosis; prevention of interferon regulatory factor 3 (IRF3)-dependent apoptosis; enhancement of PEL cell survival by c-Myc activation; activation of HIF-1α and VEGF	[195–204]
vIRF4	inhibition of Notch signalling by binding to CSL/CBF1; contribution to Rta-mediated lytic viral gene expression	promotion of p53 degradation by targeting Mdm2 and USP7	[205–209]
vMIP I–III	vMIP-I (vCCL1): induction of angiogenesis via CCR8 activation; vMIP-II (vCCL2): inhibition of NK cells migration by modulation of CCR1-5/8 and CXCR4 activity; vMIP-I and -II promote KSHV lytic reactivation; vMIP-III (vCCL3): stimulation of angiogenesis and TH2 cells attraction via CCR4 and XCR1 activation	contribution to neutrophil and TH2 cell infiltration into KS lesions and to KSHV-promoted angiogenesis; promotion of PEL cell survival by vMIP-I and -II	[210–215]

In a murine model of KS based on a mouse endothelial cell line transfected with a recombinant BAC genome, the loss of the viral genome or suppression of vGPCR (table 1) by small interfering RNA (siRNA), was associated with a loss in tumourigenicity in mice [155]. Together, these observations probably argue against a single ‘transformation event’ as tumour-initiating mechanism and in favour of a combination of KSHV-dependent effects on endothelial cell differentiation and proliferation with local inflammatory processes (see below for details). However, there is also evidence, summarized below, that KSHV has the potential to transform cells to independent growth. In the view of this heterogeneity of KS lesions, it appears possible that KS tumours differ with regard to their dependence on KSHV-induced effects and that in some a transforming event such as virus-induced DNA damage may have led to autonomously growing cell populations. This could potentially explain the variable response to currently used treatments (see below).

In the case of PEL, for which an experimental *in vitro* model is available in the form of PEL-derived lymphoma cell lines, silencing of the latent KSHV genes encoding the major latency protein LANA, vCYC and vFLIP [137,138], as well as the interferon regulatory factor homologue vIRF3 [195] inhibits the growth of PEL cell lines in tissue culture as well as (in the case of vFLIP) in a mouse xenograft model of PEL. The role of vFLIP in the survival of PEL cells may be linked to its ability to activate the NF-κB pathway, because a small molecule NF-κB inhibitor induces apoptosis in PEL cell lines [222]. Together, these observations suggest that the continued presence of the KSHV genome (mediated by LANA) and expression of the above proteins (which are part of the latency programme in B-cells) are required for the survival of PEL cells (further references in table 1). Similarly, inhibition of STAT3 signalling, which is activated by the KSHV interleukin 6 (IL6) homologue vIL6, results in apoptosis of PEL cells [223,224]. vIL6, although not considered a latent gene, is expressed in a substantial number of PEL cells [216].

Furthermore, mice double-transgenic for the KSHV latent locus (LANA, vCYC, K13, all KSHV miRNAs including the viral miR-155 orthologue, K12) and *c-myc* developed lymphoma at an increased rate [225].

No similar experimental evidence is available for KSHV-associated MCD, because of the lack of a suitable *in vitro* or *in vivo* experimental model. However, the clinical severity of MCD correlates with KSHV viral load in peripheral blood [167,226,227] as do the levels of cellular and viral IL6 and IL10 [167,228]. In addition, treatment attempts in individual cases with ganciclovir or valganciclovir [229], as well as a clinical trial using high-dose zidovudine and valganciclovir [230], reported the improvement of clinical symptoms accompanied by a decrease in KSHV viral load in peripheral blood.

### (b) KSHV induces endothelial cell proliferation

In tissue culture, KSHV-infected primary human endothelial cells show evidence of increased proliferation after longer culture and a moderately extended lifespan, but do not outgrow their uninfected counterparts in the same culture, do not become fully transformed and lose the KSHV genomes after extended *in vitro* passage [89,130,231,232]. However, evidence of transformation (growth in soft agar and under low serum conditions, tumour formation in nude mice), could be seen in a telomerase-immortalized human endothelial cell line [233] and in primary rat mesenchymal precursor cells [234] infected with KSHV, as well as in a murine endothelial cell line transfected with a recombinant KSHV genome [155]. Whether KSHV-infected endothelial cells in KS tumours *in vivo* actually proliferate at an increased rate has however not been clearly established: in one study only 20% of KS spindle cells stained positive for the cellular proliferation marker PCNA [235], while an increased staining for TP53 and Ki-67, another proliferation marker, was reported in advanced KS lesions by another group [236]. This variability may reflect the fact that KS tumours differ with regard to their staining for LANA (figure 1) and their KSHV gene expression pattern [218].

**Figure 2.**
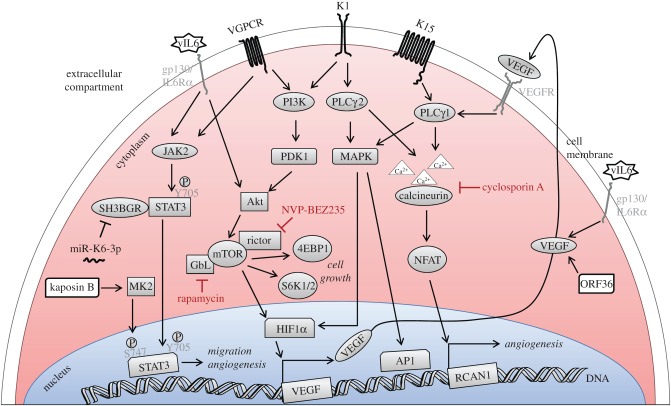
Schematic of KSHV-promoted activation of STAT3, PI3 K/Akt, MAPK and PLCγ/NFAT pathways contributing to proliferation and migration of KSHV-infected endothelial cells. The transmembrane viral proteins vGPCR, K1 and K15 as well as the viral IL6 homologue vIL6, kaposin B and ORF36 activate PLCγ, Akt, MAPK and STAT3 pathways. Some of these could be promising targets to inhibit the development of Kaposi's sarcoma.

Infection of primary human endothelial cells by KSHV activates the PI3 K/Akt/mTOR pathway. Several viral proteins, including K1, vIL6 and vGPCR have been shown to contribute to this activation [71,156,157,237]. A downstream target of this pathway, mTORC1, is activated in KSHV-infected lymphatic endothelial cells whose growth is inhibited by rapamycin, an mTOR inhibitor [238]. Interestingly, rapamycin has been shown to induce the regression of KS tumours that had developed in transplant recipients on an immunosuppressive regimen containing the cyclophilin inhibitor cyclosporin [239], suggesting that the PI3 K/Akt/mTOR pathway might represent a promising target to interfere with the proliferation of KSHV-infected endothelial cells and to inhibit the development of KS. This is illustrated schematically in figure 2. In addition to K1, vIL6 and vGPCR, several other KSHV proteins probably contribute to KSHV-induced cell proliferation. Among these are the three viral latent proteins LANA, vCYC and vFLIP, as well as the neighbouring cluster of latently expressed viral miRNAs and the viral interferon regulatory factor vIRF1 (table 1).

### (c) KSHV alters the differentiation of infected endothelial cells

**Figure 3.**
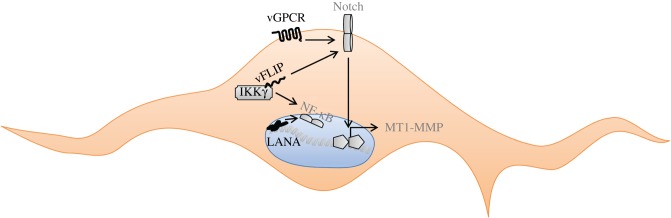
KSHV-induced activation of NF-κB and Notch signalling pathways involved in atypical spindle cell differentiation and survival. The viral proteins vFLIP and vGPCR mediate the atypical differentiation of endothelial cells by triggering the Notch signalling pathway; in addition, the vFLIP–IKKγ interaction activates the pro-survival NF-κB pathway.

Long before the discovery of KSHV, it had been noted that the histological hallmark of advanced KS lesions, the elongated spindle cell, could display markers of both endothelial (e.g. podoplanin, VEGFR3, CD34) and mesenchymal (e.g. vimentin, PDGFRα) differentiation and its cellular origin was therefore controversial [240]. This can now be explained by the finding that KSHV can change the differentiation of blood vascular endothelial cells into lymphatic endothelial cells and vice versa [241,242] and also induce the aberrant expression of mesenchymal markers, referred to as ‘endothelial-to-mesenchymal transition’, EndMT [139,243]. Similar to the epithelial-to-mesenchymal transition frequently observed in some epithelial cancers, this differentiation towards a mesenchymal phenotype is accompanied by an increased invasiveness of KSHV-infected endothelial cells (figure 3), which is mediated by the viral proteins vFLIP and vGPCR and the cellular metalloproteinase MMT1-MMP and involves the cellular Notch pathway [139,243]. The latent viral protein vFLIP is also required for the development of the characteristic endothelial spindle cells found in advanced KS tumours [140–142]. The ability of KSHV to modulate blood vascular versus lymphatic endothelial cell differentiation involves the increased expression of Prox-1, an important regulator of lymphatic endothelial cell differentiation, as well as of inflammatory and angiogenic cytokines and their receptors [241,242]. In addition, activation of STAT3 signalling and the Akt pathway by the KSHV viral IL6 homologue vIL6 has been shown to contribute to the KSHV-induced lymphatic reprogramming of endothelial cells [168,237]. KSHV-infected lymphatic endothelial cells display a more ‘relaxed’ viral gene expression pattern compared with infected vascular endothelial cells [238]. This ‘relaxed’ latency transcriptome pattern involves the expression of several viral genes that have been linked to pathogenesis, e.g. K1, K15, vGPCR (table 1), some of which have also been shown to be expressed in KS tumours [218].

### (d) KSHV promotes aberrant angiogenesis

In addition to promoting the proliferation of infected endothelial cells and altering their differentiation, KSHV can also confer additional angiogenic features on endothelial cells, which may explain some of the histological features of KS tumours, such as dilated abnormal vessels with thinned endothelium, leakage, erythrocyte extravasation and the presence of inflammatory cells (figure 1). In tissue culture, KSHV-infected primary endothelial cells form tubular structures on Matrigel in the absence of added growth factors, mimicking the effects of VEGF on (uninfected) primary endothelial cells. KSHV-infected primary and immortalized endothelial cells are also more invasive and migrate faster than their uninfected counterparts [89,90,139,232,244,245]. In addition, an increased secretion or expression of pro-angiogenic cytokines such as VEGF, Ang2, Il6, IL8, of several metalloproteinases, as well as of angiogenesis-promoting signalling components such as ephrin B2, emmprin, Hey1, PDGFRβ, c-kit has been noted [143,241,244–249]. KSHV-infected lymphatic endothelial cells grown in spheroids ‘sprout’ from the spheroid surface, reflecting their increased invasive potential [139].

### (e) The involvement of DNA damage and repair in KSHV pathogenesis

The cellular DNA damage response/repair (DDR) machinery secures the genome integrity and the transmission of intact genomes to daughter cells during mitosis. As reviewed elsewhere in this series of articles [250], the activation of specific DNA damage sensors leads to the recruitment of downstream components of the DNA repair machinery, such as the kinases ATM, ATR, p38MAPK, MK2, Chk1 and Chk2, as well as the transcription factor p53, whose main function is to slow down the cell cycle and thereby allow the repair of the damaged DNA, or to induce apoptosis to avoid the propagation of a mutated genome. Activation of the DDR machinery in response to intracellular signalling pathways triggered by cellular oncogenes leads to cell cycle arrest and ‘oncogene-induced senescence’ (OIS) and therefore has a tumour-suppressing role. As several DNA viruses activate intracellular signalling pathways that are also triggered by cellular oncogenes, they have evolved mechanisms to antagonize the host DDR machinery to prevent these intracellular defence mechanisms from inducing OIS and thereby curtailing the replication of viral genomes (see accompanying review by M. Weitzman and colleagues [250]).

**Figure 4.**
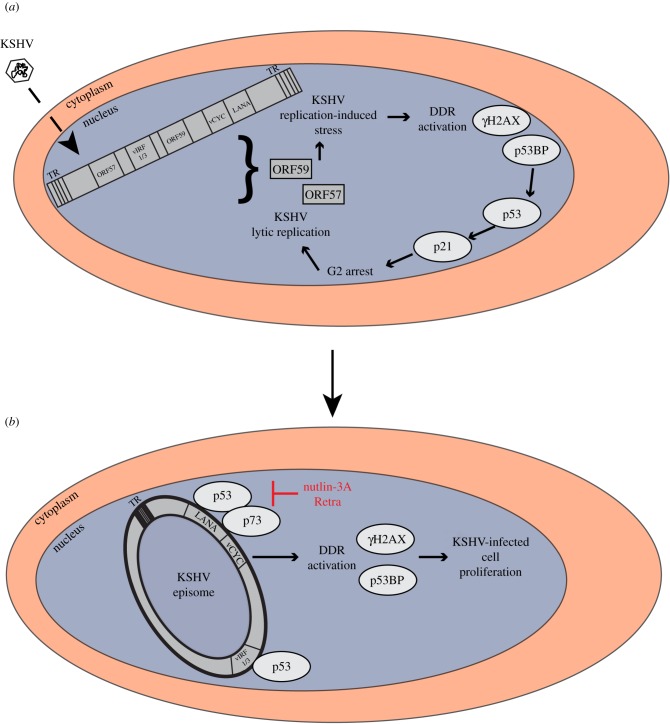
The role of the DDR machinery in KSHV lytic (*a*) and latent (*b*) replication. DDR signalling induced by the sensing of viral DNA leads to a cell cycle block and ultimately favours KSHV lytic replication (*a*). Subsequently, the neutralization of p53 and p73 activity by viral proteins, e.g. LANA, removes the cell cycle block and allows KSHV latent replication (*b*). TR, terminal repeats.

Infection of telomerase-immortalized human primary endothelial cells by KSHV initially leads to a p53-dependent growth arrest, which may be triggered by the action of the KSHV viral D-cyclin homologue vCYC; expressed on its own, vCYC can induce a DNA damage response and p53-dependent growth arrest of vCYC-expressing cells [130]. After extended passage of KSHV-infected endothelial cells in tissue culture, this growth arrest is overcome and their subsequent proliferation depends on the blocking of p53-dependent apoptosis pathways by viral proteins [130]. The proliferation of KSHV-infected endothelial cells or PEL cell lines can be inhibited by the small molecule inhibitor nutlin-3a, which antagonizes the p53 ubiquitin ligase Mdm2 and thereby restores p53 function [251]. Similarly, the small molecule inhibitor retra, which restores the function of the p53 homologue p73 in cells with a mutated p53, can induce apoptosis in p53 mutant PEL cell lines [103]. As nutlin-3a and retra can inhibit the binding of the major KSHV latency protein LANA to, respectively, p53 and p73, as well as the ability of LANA to antagonize p53- or p73-dependent transcriptional activation [103,251], it is likely that LANA is one of the viral proteins required for neutralizing the p53-dependent growth arrest [252–254], which is initially observed in KSHV-infected endothelial cells (figure 4). In PEL cells, the function of p53 or p73 can be restored, and apoptosis induced, either by disrupting the binding of p53 to LANA [103,251] or by silencing LANA by siRNA [137,138]. In addition, other KSHV proteins shown to interact with p53 and to antagonize its function, such as the viral interferon regulatory factor homologues vIRF1 and vIRF3, may be important in this context [195,196,255]. vIRF3, also termed LANA2, is expressed in latently infected B-cells, in particular PEL cells [196,256] and its silencing by siRNA in PEL cell lines induces a growth arrest [195].

During latent infection of cultured peripheral blood mononuclear or primary endothelial cells, γH2AX, the phosphorylated form of the histone variant H2AX and a key molecule in the DNA damage repair cascade activation, co-localizes with LANA and the KSHV genome [130,257,258]. In KS tumours, a proportion of KSHV-infected endothelial spindle cells show evidence of an activated DDR response (γH2AX and p53BP staining), particularly in early stage lesions [130]. Phosphorylation of H2AX may directly benefit KSHV replication or latent persistence. In analogy to the finding that the related γ-herpesviruses EBV and MHV-68 can directly phosphorylate H2AX by means of their ORF36/BGLF4 kinases and that ORF36-mediated phosphorylation is required for efficient MHV-68 replication [259], H2AX has been shown to be phosphorylated in KSHV vCYC- or LANA-transfected cells, and γH2AX to be deposited on the KSHV latent origin of replication and to be required for latent episome persistence [130,257,258]. Other DDR components to be recruited by LANA include the nuclear uracil-DNA glycosylase UNG2, which is involved in base excision repair. UNG2 depletion in PEL cells leads to a reduced number of viral genome copies and to a production of infection-deficient virus [260]. The LANA-mediated recruitment of ubiquitin-specific protease 7 (USP7), involved in the transcription-coupled nucleotide excision repair pathway, modulates KSHV latent replication [261]. The Ku70/Ku86 heterodimer phosphorylates LANA and thereby impairs LANA-dependent KSHV latent replication [262]. Together, these findings highlight the involvement of DDR proteins in the establishment and maintenance of KSHV latent infection.

A strong DDR response is also triggered by KSHV lytic replication [263]. The KSHV lytic cycle protein encoded by ORF57 promotes the nuclear export and translation of viral mRNAs at the expense of cellular mRNAs by recruiting the human transcription and export complex and thereby causes an aberrant accumulation of cellular newly transcribed mRNA in the so-called unstable R-loops which can cause double-strand breaks in cellular DNA [264]. In addition, another KSHV lytic protein, ORF59 or PF-8 (processivity factor-8), a DNA-binding protein supporting viral DNA synthesis and infectious virus production, has been reported to impair non-homologous end-joining repair activation by binding to the Ku70/Ku86 heterodimer, thereby inhibiting the subsequent recruitment of DNA-dependent protein kinases and causing double-strand break formation [265]. Activation of p53-dependent effectors, in particular transcription of the Cdk inhibitor p21, appears to be required for efficient lytic replication by arresting cells in the G2 phase of the cell cycle and thereby either providing access to the DDR machinery or preventing the condensation of chromatin on the viral genome during mitosis [266]. In line with the latter interpretation, TLK kinases, known to modulate the entry into mitosis and chromatin condensation, have been shown to suppress KSHV lytic replication [267].

These observations indicate that KSHV latent persistence as well as lytic replication may benefit from the activation of DDR pathways. Figure 4 shows a model that attempts to connect these observations. This model suggests that the DDR induced early after virus entry by the sensing of incoming viral DNA, activation of ATM [258] and perhaps the expression of vCYC [130] would lead to a p53-dependent cell cycle block that would favour lytic (productive) viral replication [266]. The subsequent neutralization of p53 and p73 by LANA and (in B-cells) vIRF3 would remove the cell cycle block and allow entry into latency, during which duplication of the viral episome happens during S phase and replicated episomes are partitioned to daughter cells during mitosis. Disruption, during latency, of the LANA-p53/p73 complex by the small molecule inhibitors nutlin-3a or retra restores a p53/p73-dependent cell cycle block and thereby lytic reactivation [266] (G Mariggiò, S Santag 2012, unpublished data). There may, however, also be an impact of the activated DDR on cellular DNA, as chromosomal abnormalities, in particular centrosome amplifications, have been noted in KSHV-infected and vCYC-overexpressing cultured endothelial cells [130,268,269].

### (f) DDR response and innate immunity

As illustrated by the above examples, the activation of DDR signalling cascades by viral infections can be viewed as part of an ‘intrinsic’ intracellular defence against viral DNA. This role of the DDR is connected to ‘conventional’ mechanisms of innate immunity [270,271]. Here, the detection of ‘foreign’ DNA in the cytoplasm and/or nucleus by the DDR components Rad50, Mre11, interferon γ-inducible protein 16 (IFI16) or DNA-dependent protein kinases leads to the activation of innate immune response pathways such as the activation of interferon regulatory factor 3 (IRF3) and NF-κB, followed by the downstream expression of chemokines and inflammatory cytokines [104,272–282].

In the case of KSHV, the innate nuclear DNA sensor IFI16 recognizes latent viral episomes in a complex with the DNA damage repair sensor BRCA1 and induces the inflammasome and interferon β responses [280,281]. During lytic replication, IFI16 inhibits the expression of lytic viral genes but is itself degraded by late viral proteins to allow progression of the lytic replication cycle [282].

More recently, it has been reported that a sensor of cellular DNA, cGAS, is antagonized by several KSHV proteins, including the viral kinase encoded by ORF36, LANA, ORF57, vIRF1, ORF45, ORF52 and ORF55 [104,183,283,284]. A cytoplasmic form of LANA [285] directly interacts with cytoplasmic cGAS to restrict the cGAS-dependent activation of STING, TBK1, IRF3 and the induction of interferon gene expression in order to facilitate reactivation of KSHV from latency [104]. In an extension of this concept, we have recently found that the cellular DNA damage sensors Rad50 and Mre11, which are known to activate the STING/interferon and NF-κB pathways in response to cytoplasmic DNA [276,278], are also antagonized by cytoplasmic LANA variants to promote lytic reactivation [286].

### (g) KSHV induces and benefits from inflammation

Several clinical observations, as well as histological and experimental evidence, suggest that KSHV reactivation is accompanied by inflammatory processes and that these may contribute to its pathology. KS tumours can arise in traumatized skin areas (Koebner phenomenon) [287], indicating that wound repair mechanisms support the development of this tumour. Inflammatory cellular infiltrates, consisting of monocytes, eosinophils and plasma cells, are frequently seen in KS lesions [288]. TH1 cytokines and interferons increase the KSHV viral load in peripheral blood B-cells and monocytes [289,290]. In HIV-infected patients, KS can develop within a few months after the start of combination antiretroviral therapy (cART), as part of the ‘immune reconstitution inflammatory syndrome’ (IRIS) [291–293].

In culture, KSHV-infected primary endothelial cells show an increased expression of inflammatory and angiogenic cytokines, such as IL6, IL8, IL15, IL16 and several chemokines [89,141,241], in addition to the viral IL6 homologue vIL6 and the three viral chemokines vCCL1–3. In KS lesions, a few KSHV-infected cells express vIL6 [216,217]. As mentioned earlier, vIL6 and vIL6-induced STAT3 activation are thought to play a role in the KSHV-induced lymphatic differentiation of infected vascular endothelial cells [168,237]. In addition, vIL6 is expressed in PEL and MCD, and vIL6, via vIL6-induced STAT3 activation, plays a crucial role in the continued proliferation of PEL cells (see above). In MCD, the variation of clinical symptoms in individual patients, as well as their KSHV viral load, is often mirrored by fluctuating vIL6, IL6 and IL10 levels [167,226–228].

The cellular inflammation mediator cyclooxygenase 2 (Cox-2) is expressed in KS tumours and induced by KSHV in infected cultured endothelial cells [245,294] through the action of several viral proteins, including vFLIP and K15 (table 1). The increased expression of Cox-2 in KSHV-infected endothelial cells contributes to the secretion of cellular chemokines (RANTES, MCP2, TARC, MIP1α, MDC) that modulate leucocyte trafficking and of pro-angiogenic factors (IGF1, PDGF, IL14, MCSF, GM-CSF, VEGF A, VEGF C, angiogenin, oncostatin M, TGFβ1); Cox-2 also enhances the activation of KSHV in latently infected endothelial cells as well as their invasiveness [245,294]. However, although a number of inflammatory signalling components have thus been experimentally linked to KSHV reactivation, their individual contribution to the development of KS remains to be established.

### (h) Other cofactors contributing to disease development

In addition to the contribution of immune suppression and inflammation to KSHV pathogenesis (see above) it is very likely that other, as yet unidentified, cofactors play a role in disease development. For example, the much higher rates of classic KS in males, originally noted by M. Kaposi, still await a satisfactory explanation, as KSHV seroprevalence in southern Europe does not differ between men and women in most studies [21].

Genetic predisposition can increase the risk for a KSHV-infected individual to develop KS. Several cases of the extremely rare form of classic KS in children have been reported in consanguineous families and mutations in the genes for IFNR1, STIM, OX40/CD134 and WAS have been shown to be the likely underlying genetic cause [295–299]. The genetic defects all appear to compromise an efficient T-cell response to KSHV, indicating that genetically determined, as well as iatrogenic (for post-transplant KS) and infection-related (for AIDS KS) T-cell defects can predispose to KS development. In addition, a mutation altering the protein sequence of STAT4 was identified in a family in which five members, most of them women, developed classic KS at an advanced age [300]. Additional families with a high penetrance of classic KS have also been reported [301], again suggesting an underlying genetic contribution. In case–control studies, particular genetic variants of the genes encoding FCGR3A, CXCR2 and IL13 have been found to be associated with classic KS [302,303], although the functional significance of these variants has not yet been established. Altogether, these studies strongly indicate that genetically determined immunodeficiencies can predispose to the development of KS in a KSHV-infected individual at an early age and that other cellular genes may act as ‘disease modifiers’. There are thus interesting parallels to X-linked immunoproliferative disease following a primary Epein–Barr virus infection.

## 4. Implications for therapy and prevention

KSHV-associated diseases, KS, MCD and PEL, represent important and difficult to treat clinical problems. Although the success of cART has dramatically reduced the incidence of AIDS-KS in HIV-infected patients, this tumour can occur in patients on cART with low or undetectable HIV load and CD4 counts greater than 300 CD4 T-cells/mm^3^ [304]. In one report from the USA, about a third of HIV-associated KS cases still occurred in such a group of successfully treated HIV patients [305]. AIDS-KS is also seen as a first clinical symptom in patients with undiagnosed HIV infection and in advanced AIDS associated with cART failure. AIDS-KS can flare up after the initiation of cART as part of the IRIS (see above). In the transplant setting, KS is a significant cause of morbidity and mortality in countries with a higher KSHV prevalence (see above), e.g. in Italy, Turkey and Saudi Arabia. Transplant KS appears to be more common in kidney transplant recipients and has been estimated to occur in approximately 0.5–5% of solid organ transplant recipients, depending on the geographical region [18]. Its clinical severity ranges from isolated skin tumours to rapidly progressing visceral involvement in conjunction with severe haematological abnormalities such as thrombocytopenia, anaemia and bone marrow failure [18]. Lastly, endemic KS in sub-Saharan Africa, where it often occurs in children, awaits satisfactory treatment options.

Many of the treatments for KSHV-associated diseases were developed before the discovery of KSHV as the key aetiological agent. Recently, however, our increasing understanding of its role in pathogenesis has enabled the design of new treatment strategies, which target either KSHV itself, particular infected cells or key cellular pathways that have been shown to be important in KSHV biology.

Long established treatment options are based on cancer chemotherapy and involve the use of liposomal formulations of pegylated doxorubicin or daunorubicin, vincristine, bleomycin and etoposide as single agents or in combination, with taxol as a second line therapy option [306,307]. Radiotherapy or surgery is also sometimes used for isolated lesions. Reduction of immune suppression in transplant recipients will often improve post-transplant KS, and cART has made a huge impact on the incidence of AIDS-KS, with the exceptions noted above.

The only currently available ‘directly acting antivirals’ against KSHV are inhibitors of the KSHV DNA polymerase which were originally developed against other herpesviruses; those with activity against KSHV include ganciclovir, cidofovir and foscarnet [308–311]. Valganciclovir has been shown to reduce KSHV shedding in saliva of KSHV-infected individuals [312]. In KSHV-infected patients ganciclovir and foscarnet may lower the KSHV viral load in peripheral blood and occasionally improve KSHV-associated MCD or haemophagocytic syndrome [13,229,313,314]. Prophylactic treatment with ganciclovir for CMV disease is associated with a reduced incidence of KS in AIDS patients [315].

A combination of oral high-dose zidovudine and valganciclovir, which are, respectively, phosphorylated and thereby activated to become cytotoxic by the KSHV thymidine kinase (ORF21) and protein kinase (ORF36), achieved a high rate of clinical responses and a lowering of the inflammatory laboratory parameters (C-reactive protein, IL6, IL10) and KSHV viral load in HIV-infected patients with MCD [230]. While the success of this treatment regimen may be based on the activation of cytotoxic prodrugs in, and subsequent killing of, KSHV-infected B-cells, it is also conceivable that the inhibition of KSHV lytic replication by activated valganciclovir may also have contributed [230]. Rituximab, a monoclonal antibody to the B-cell surface protein CD20, has been used successfully, alone or in combination with liposomal daunorubicin, for the treatment of MCD in AIDS patients [316–319]. However, the use of rituximab alone has been reported to worsen KS, which is frequently found in patients with MCD [318]. Rituximab has also been found to be effective against PEL [320].

An example of a new treatment option based on a better understanding of KSHV pathogenesis is the observation that switching patients with post-transplant KS to an immunosuppressive regimen containing rapamycin can control their KS lesions [239,321]. Rapamycin binds to FK506-binding protein 12 (FKB12) and the rapamycin-FKB12 complex inhibits the activity of the cellular mTOR kinase [322] in the PI3 K/Akt pathway. As reviewed above, this pathway is now known to contribute to the proliferation and angiogenic properties of KSHV-infected endothelial cells (figure 2). Furthermore, the appreciation of the importance of receptor tyrosine kinases, such as PDGFR and c-kit in the life cycle of KSHV has led to clinical trials exploring the efficacy of imatinib, an inhibitor of Abl as well as of these two tyrosine kinases, against KS; these trials have reported a partial regression of KS tumours in about a third of treated patients [323,324]. Similarly, sorafenib, which targets receptors of VEGF and PDGF, has been tried [325]. Following the report that the KSHV thymidine kinase encoded by ORF21 serves as a protein tyrosine kinase [326], we recently showed that its tyrosine kinase activity is required for an efficient activation of the lytic replication cycle and identified additional US Food and Drug Administration approved tyrosine kinase inhibitors that potently inhibit KSHV lytic replication (G Beauclair, T Dubich, E Naimo, J Rückert, S Koch, A Dhingra, D Wirth, TF Schulz 2017, unpublished data).

With regard to prevention, from an epidemiological point of view KSHV seems to represent a case of a virus whose transmission should be preventable by vaccination. At least in middle-to-high-income countries, it is preferentially found in certain geographical regions or particular groups at risk for KSHV transmission (see above) and a vaccine could therefore be directed at specific populations. So far, only one successful vaccine for a herpesvirus (varicella zoster virus) is available. The success of this vaccine should stimulate efforts to develop an efficacious vaccine against KSHV. In order to be a candidate vaccine, it would have to be able to prevent primary infection, as KSHV-associated diseases, including clinical manifestations of primary infections, are too rare to be used as endpoints in a vaccine trial. Unfortunately, attempts to develop vaccines against the related γ-herpesvirus, Epstein-Barr virus have so far been limited, with only a gp350-based vaccine showing potential efficacy against the clinical manifestation of infectious mononucleosis, but not primary infection [327]. It is likely that novel approaches, such as the use of virus-like particles, will be necessary to develop an effective KSHV vaccine.

## 5. Conclusion and outlook

Nearly 150 years after the first description of KS and 22 years after the discovery of KSHV, the efforts of many investigators have laid the foundations for a much improved understanding of this virus and its role in the diseases that it is now known to be associated with. As in the case of its taxonomically close relative, Epstein–Barr virus, genetic, environmental or infectious cofactors are required for disease development. In the case of KSHV, inflammation and an insufficient T-cell surveillance, due to genetic predisposition, iatrogenic immune suppression or co-infections appear to be the most important cofactors. Our improved understanding of KSHV pathogenesis should now allow us to focus on key viral and cellular modulators of the viral life cycle in order to develop improved therapeutic modalities. Of particular interest could be the early phase of the lytic replication cycle, which appears to be adopted by the virus in lymphatic endothelial cells and in many KS biopsies, as well as in MCD and some PEL tumours, and during which key viral proteins with pathogenic properties are expressed. Targeting the activation of key cellular signalling cascades by one or several of these viral proteins could represent a promising approach to curtail the pathogenic effects of KSHV. In addition, the latency programme of KSHV, in particular LANA, which is essential for viral persistence, could represent a therapeutic target that would be worth pursuing. In view of the unusual distribution of KSHV and its potential for sexual transmission, there is probably a case for developing a KSHV vaccine that could be directed at groups who are at an increased risk of contracting this virus.
